# Are hepatic steatosis and carotid intima media thickness associated in obese patients with normal or slightly elevated gamma-glutamyl-transferase?

**DOI:** 10.1186/1479-5876-10-50

**Published:** 2012-03-16

**Authors:** Giovanni Tarantino, Carmine Finelli, Annamaria Colao, Domenico Capone, Marianna Tarantino, Ernesto Grimaldi, Donato Chianese, Saverio Gioia, Fabrizio Pasanisi, Franco Contaldo, Francesco Scopacasa, Silvia Savastano

**Affiliations:** 1 Department of Clinical and Experimental Medicine, Federico II University Medical School of Naples, Naples, Italy; 2 Fondazione Stella Maris Mediterraneo, Centro di Riferimento Regionale dei Disturbi e Comportamenti Alimentari e del Peso, "G. Gioia", Chiaromonte, (PZ), Italy; 3 Department of Molecular and Clinical Endocrinology and Oncology, Endocrinology Section, Federico II University Medical School of Naples, Naples, Italy; 4 Department of Neurosciences, Unit of Clinical Pharmacology, Federico II University Medical School of Naples, Naples, Italy; 5 Department of Biomorphological and Functional Sciences, Federico II University Medical School of Naples, Naples, Italy; 6 Department of Biochemistry and Medical Biotechnology, Federico II University Medical School of Naples, Naples, Italy

**Keywords:** NAFLD, Atherosclerosis, Metabolic syndrome

## Abstract

**Background:**

Hepatic steatosis (HS) has been associated with obesity and metabolic syndrome (MS), conditions carrying a high risk of coronary artery disease. We aimed to determine whether HS was an independent factor of atherogenic risk beyond its association with MS and its components.

**Methods:**

We assessed the circulating levels of the heat shock protein-70 (HSP-70), a chaperone involved in inflammation, endoplasmic reticulum stress and apoptosis at liver and endothelial level and the gamma-glutamyl transferase activity (γ-GT) correlating them to carotid intima-media thickness (IMT), along with lipid profile, HOMA, C-reactive protein, fibrinogen, ferritin, adiposity type as well as spleen volume in 52 obese pts with grade 1, 128 with grade 2, and 20 with grade 3 of HS evaluated by sonography.

**Results:**

Patients with different grade of HS demonstrated overlapping HSP-70 levels; similarly performed obese subjects regarding IMT. Using multiple regression analysis, IMT was predicted by age, visceral adiposity and by HOMA (β = 0.50, *p *< 0.0001, β = 0.30, *p *= 0.01 and β = 0.18, *p *= 0.048 respectively, while the severity of HS was predicted by visceral and subcutaneous adiposity and HOMA (β = 0.50, *p *< 0.0001 and β = 0.27, *p *= 0.001 and β = 0.18, *p *= 0.024, respectively).

**Conclusion:**

In our series of patients with normal or mild elevation of γ-GT, the severity of HS does not entail higher IMT, which may be linked to MS stigmata.

## Background

Unclassified nonalcoholic fatty liver disease (NALFD) or hepatic steatosis (HS), a further expression of the metabolic syndrome (MS) [1], easily detected by ultrasound (US), is highly prevalent condition in obese individuals. Free fatty acids (FFAs) have been shown to be the major contributor to triglyceride accumulation in hepatocytes observed in NAFLD [2]. The excessive supply of FFAs toward the liver leads *per se *to hepatic insulin resistance (IR) and endoplasmic reticulum stress (ERS) [3]. Heat shock proteins (HSPs) function as intra-cellular chaperones. The unfolded protein response (UPR), a fundamental cellular process triggered by ERS, is aimed at initiating programmed cell death. The UPR is activated and dysregulated in NAFLD [4]. On the other hand, chronic ERS activates UPR in arterial endothelium in regions of susceptibility to atherosclerosiss [5]. ERS activation participates in fat deposition in the liver [6] and could directly induce IR. Insulin-resistant state in turns increases the plasma FFAs flux [7]. The best known member of HSP is the stress-inducible form of HSP-70, i.e., HSP-72, also called HSPA1A. The HSP-70 expression decreases with age in humans [8]. Hamsters fed high-fructose diet exhibit fat accumulation in liver and the Hsp70 expression is down-regulated [9]. These data were confirmed in obese Zucker rats with HS [10]. Ischemic preconditioning by increasing HSP-72 protects steatosic livers [11]. In wild-type mice, refractory to high-fat dietary-induced effects, a marked increase in HSP-72 levels in liver was evidenced [12]. HSP-70 can be produced in the liver and spleen as acute phase reactant and released into circulation to facilitate the disposal of dying cells [13]. Low-grade chronic inflammation, which is characterized by increased serum concentrations of interleukin-6 (IL-6) and enlarged spleen volume [14] likely due to dendritic cells (DCs) mobilization, is contributing factor in developing the more severe form of HS in obese patients. HSPs have been reported to play important roles in activation and maturation of DCs [15]. Moreover, the concentration of HSP-70 is inversely correlated to IL-6 [16]. Gamma-glutamyltransferase (γ-GT) is a subclinical clue of IR [17], even though the prevailing interpretation is that its high serum levels represent just an early evidence of oxidative stress. Its mechanism relies on the fact that cellular γ-GT is closely linked to metabolism of glutathione (GSH), the most abundant intracellular antioxidant [18]. Depletion of GSH affects the synthesis of HSP-70 in Hep G2 cells [19]. Finally, HS is characterized by elevated levels of γ-GT [20], which is also a predictor of coronary artery disease (CAD) [21].

The direct contribute of HS to early atherosclerosis, evaluated as increased intima media thickness (IMT), is controversial. Against this background, we thought of exploring the behavior of HSP-70 and γ-GT, molecules playing a key role in both liver and endothelium, in obese patients with different entity of hepatic fat storage in relationship to carotid IMT in order to establish whether NAFLD was an independent factor of atherogenic risk beyond its association with MS and its components. Contextually, we tried to find out whether serum levels of HSP 70 and γ-GT correlated to metabolic indices, anthropometric measures, type of adiposity, inflammatory markers [22], and finally immune status, focusing our attention on liver-spleen axis [23].

## Methods

This cross-sectional study was performed enrolling outpatients from September 2009 through February 2011. Clinical investigation, blood samples and ultrasound (US) parameters were strictly carried out within two months. Protocol was consistent with the principles of the Declaration of Helsinki and participants gave their informed consent, according to our Medical School committee approval.

### Inclusion criteria

Two hundred obese patients with at least another criterium of those below specified clustering MS, diagnosed suffering from NAFLD by evaluating the liver/kidney difference of brightness at US, with or without elevated alanine aminotransferase (ALT) or γ-GT, were studied. They were allocated in three groups according to the severity of HS scored at US.

Obese individuals were selected to participate in this study before being given weight control indications. The degrees of obesity was established on the basis of body mass index (BMI) cut-off points of 30-34.9, 35-39.9 and >40 kg/m^2^, respectively. Central obesity was identified by waist circumference (WC) >102 cm in men or >88 cm in women, measured at the midpoint between the lower border of the rib cage and the iliac crest. Presence of type 2 diabetes mellitus (T2D) was assessed by a fasting plasma glucose ≥126 mg/dl (≥7.0 mmol/l), confirmed on a separate day or a random glucose level of 200 mg/dL or higher and classic symptoms of T2D (polyuria, polydipsia, polyphagia, weight loss) or HbA1c ≥6.5%. Impaired fasting glucose (IFG) was set with a fasting plasma glucose between 100 and 125 mg/dl. MS was defined according to the revised Adults Treatment Panel III (2001), and three or more criteria were considered: plasma glucose concentration of at least 100 mg dL-^1^, WC >102 cm in men and >88 cm in women, serum high-density lipoprotein (HDL)-cholesterol concentration <40 mg dL^-1 i^n men and <50 mg dL^-1 i^n women, blood pressure of at least 130/85 mm Hg, and serum triglyceride concentration of at least 150 mg dL ^-1^. The subjects were classified as insulin resistant according to a stringent homeostasis model assessment of IR (HOMA) setting the cut-off at 2.

Exclusion criteria were a history of previous acute CAD in course of T2D, infectious chronic diseases including hepatitis B and C, neoplastic and/or haematological diseases, autoimmune and storage diseases, unstable medical conditions, drugs inducing HS (steroids and amiodarone) and prior use of medications known to affect inflammation (aspirin), blood lipids (statins) or insulin sensibility (metformin) and finally bariatric surgery. Alcohol abuse was ruled out, according to the DSM-IV diagnostic criteria, by means of screening tests such as MAST (Michigan Alcohol Screening Test) and CAGE (Cut down, Annoyed, Guilty, and Eye opener), as well as random tests for blood alcohol concentration and the use of a surrogate marker, e.g., Mean Corpuscular Volume.

### Instrumental measurements

They were performed using GE *vivid *systems (General Electric, Milan Italy). Transverse scanning was performed to measure the subcutaneous adipose tissue (SAT) using a 11 MHz linear probe and visceral adipose tissue (VAT) using a 4 MHz convex probe. The SAT was defined as the thickness between the skin-fat interface and the linea alba, avoiding compression, and the VAT was defined as the distance between the anterior wall of the aorta and the internal face of the recto-abdominal muscle perpendicular to the aorta, measured 1 cm above the umbilicus. When the aortic walls were not visualized as they were obscured by bowel gas, the doppler scan was used.

Spleen longitudinal diameter (SLD) was performed by postero-lateral scanning [14]. The classification of severity of HS or entity of hepatic fat storage was based on the following scale of hyperechogenity: 0 = absent, 1 = light, 2 = moderate, 3 = severe, pointing out the difference between the densities of the liver and the right kidney [24]. Technically, echo intensity can be influenced by many factors, particularly by gain intensity. To avoid confounders that could modify echo intensity and thus bias the comparisons, the mean brightness levels of both liver and right kidney cortex were obtained on the same longitudinal sonographic plane. The common carotid, the carotid bulb and the internal carotid near and far wall segments were scanned bilaterally. Subjects were examined in the supine position with the head turned 45° contra-lateral to the side of scanning. Images were obtained in longitudinal section, with a single lateral angle of isonation, optimizing the image for the far wall. IMT was defined as the distance between the lumen-intima and the media-adventitia ultrasound interfaces. Measurements were performed off-line and consisted of six manual measurements at equal distances along 1 cm on the far wall of the common carotid. Left and right IMT were averaged.

The systolic/diastolic blood pressure (SBP/DBP) was the average of three consecutive readings taken by the physician during the day, during usual practice hours, and after subjects had rested for 5 min in the sitting position. During the 30-minute preceding the measurement, subjects were required to refrain from smoking or consuming caffeine. Patients on antihypertensive therapy maintained a balanced therapeutic regimen based on calcium channel blockers or ACE inhibitors throughout the study.

### Laboratory data

The HSP70 determination was made on sera stored at -70°C using a high sensitivity EIA kit by Enzo Life Sciences International, Inc., PA USA. This is the only commercially available kit sensitive enough to quantify HSP-70 in serum and plasma samples, fully validated in stressed and non-stressed serum and plasma samples and able to measure HSP-70 with negligible reactivity with other HSP family members. The absorbance was read at 450 nm with results in 4/5 hours. The sensitivity range was 0.09-12.5 ng/ml; the sensitivity or limit of detection of the assay was 0.09 ng/mL (90 pg/mL). The sensitivity was determined by interpolation at 2 standard deviations above the mean signal at background (0 ng/mL) using data from 7 standard curves. The intra-assay precision was determined by assaying 20 replicates of three buffer controls containing Hsp70 in a single assay with a CV ranging from 3.9 to 11.4%. The inter-assay precision was determined by measuring buffer controls of varying HSP-70 concentrations in multiple assays over several days with a CV ranging from 12.8% to 19.1%. Forty one apparently healthy, lean subjects, without HS at US were selected to set our normal values of serum HSP-70.

CRP values were determined by the ELISA test, with reference values between 0.3 and 8.6 mg/L in healthy men and between 0.2 and 9.1 mg/L in healthy women (BioCheck, Inc CA, USA). Total cholesterol, high-density lipoprotein (HDL) cholesterol, and triglyceride concentrations were assessed by enzymatic colorimetric methods; ALT and γ-GT by the enzymatic UV method; and glucose concentration by a hexokinase method. The fasting insulin level was measured by an immunoradiometric assay with a BioSource INS-IRMA Kit (BioSource, Belgium). Its coefficient of variation was 1.6% to 2.2% for the intra-assay and 6.1% to 6.5% for the inter-assay. Fibrinogen was determined using an ELISA kit by United States Biological, TX, USA.

Blood alcohol concentration was measured by a fluorescence polarization immunoassay technology using an Abbott kit on AxSYM Instrument, Abbott Park, Illinois.

HOMA was assessed by the formula: fasting insulin (μU/mL) × fasting glucose (mg/dL)/405.

### Statistics

Age, BMI, WC, γ-GT, ALT, cholesterol, CRP, fibrinogen, HOMA, SBP, DBP, SAT, VAT, SLD and IMT were not normally distributed when analyzed by Shapiro-Wilk (S-W) test, *p *= 0.001 and were expressed as median plus 25-75 inter-quartile range (IQR). HDL (S-W, *p *= 0.20) triglycerides (S-W, *p *= 0.44) and ferritin (S-W, *p *= 0.16) data, derived from a normally distributed population, were articulated as mean plus SD. The Mann-Whitney (M-W) *U *test for independent samples was used when managing two populations to track differences of medians. When comparing throughout the three grades of HS, the ANOVA test with Student-Newman-Keuls test for pairwise comparison of subgroups or ANOVA Kruskal-Wallis test with post-hoc analysis, Conover-Inman test, was adopted. When the ANOVA analysis was adjusted for age, BMI and WC, considering them as covariates, the ANCOVA test was applied. Pearson's chi square, was used to look for differences in the classification system. Tracking the degree of association between variables the Spearman's rho for non uniform intervals was used. To assess the independent effect of a quantitative variable on the prediction of another one, the linear regression analysis (least squares) was used. At multivariate analysis, the multiple regression (Backward Stepwise Selection) was adopted, firstly entering all variables if *p *= 0.05 into the model, and next removing if *p *= 0.1 the nonsignificant variables sequentially, with a maximum number of steps of 15. To avoid multicollinearity, i.e., situations in which the predictors are correlated to each other to some degree, the variance inflation factor and tolerance were set to >10 and <0.1, respectively. Similarly, to get sense of which variables contribute more or less to the regression equation, the magnitude of standardized coefficient beta (β) was calculated. The Factor Analysis was applied to detect the structure in the relationships among variables, selecting a subset of variables having the highest correlations with the principal component factors. The Cattell Scree plot, with relative eigenvalues, was performed to screen the real factors. Extraction of the main components amounted to a variance maximizing (varimax) the rotation of the original variable space. To evaluate intra/inter-observer variability of the measurements, the mean difference in the measurements of the observers was first calculated. Next, the concordance correlation coefficient (ρ_c_), which measures precision and accuracy, was adopted to evaluate the degree of pair observations at US, with values >0.8 being considered indicative of good reliability.

## Results

**Figure 1:**
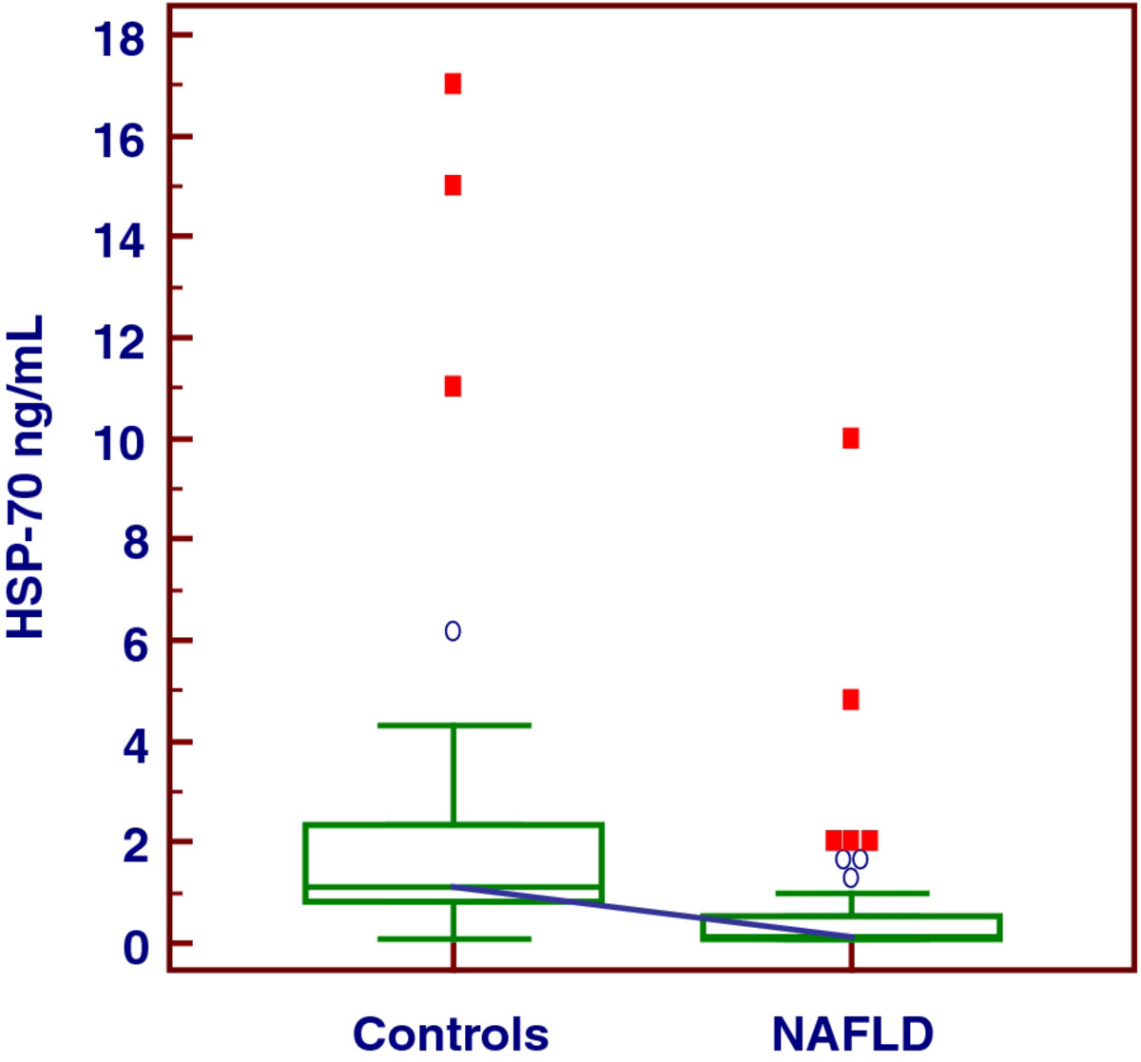
The behavior of heat shock protein-70 in Controls and obese patients.

**Figure 2:**
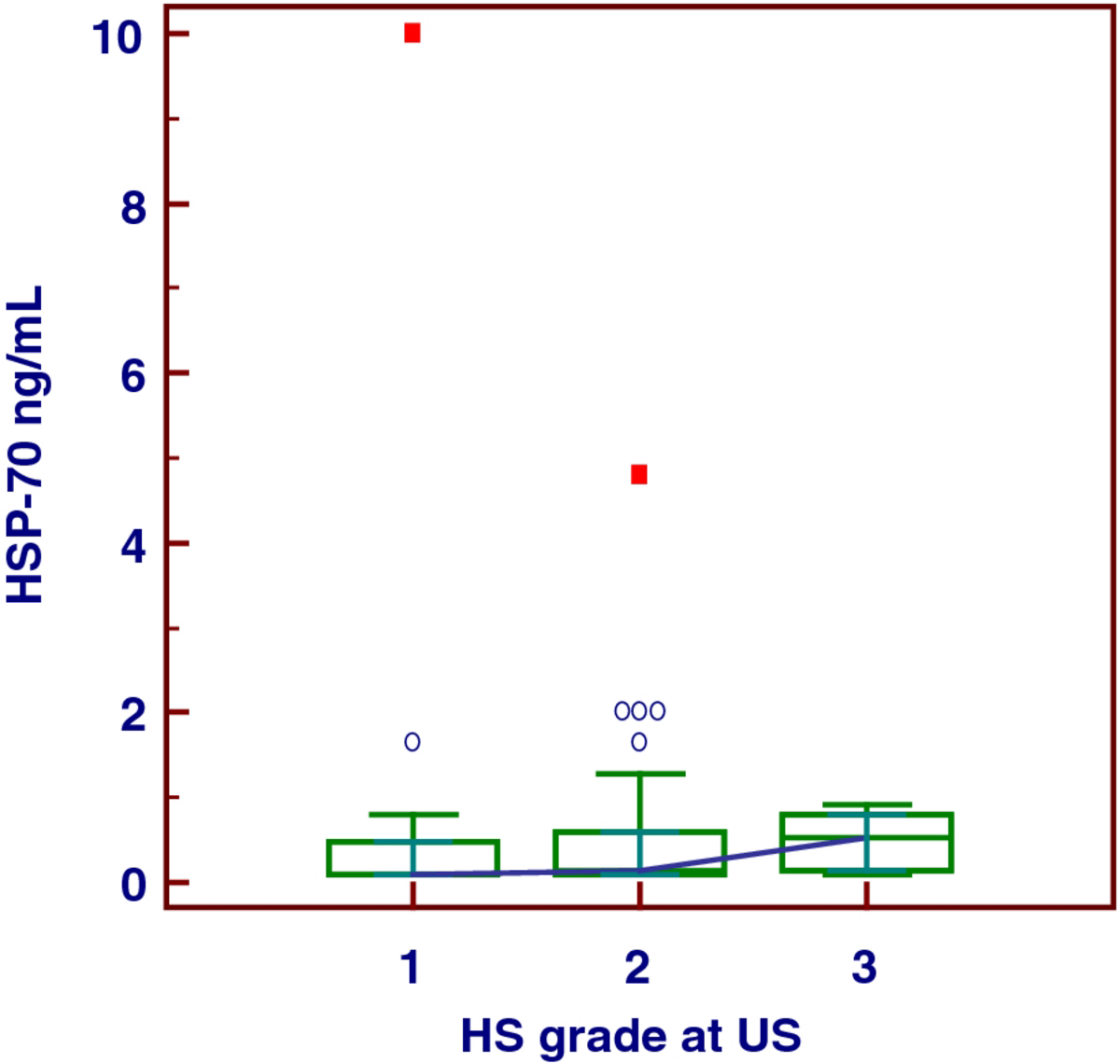
The behavior of heat shock protein-70 in obese patients with different severity of hepatic steatosis.

**Figure 3:**
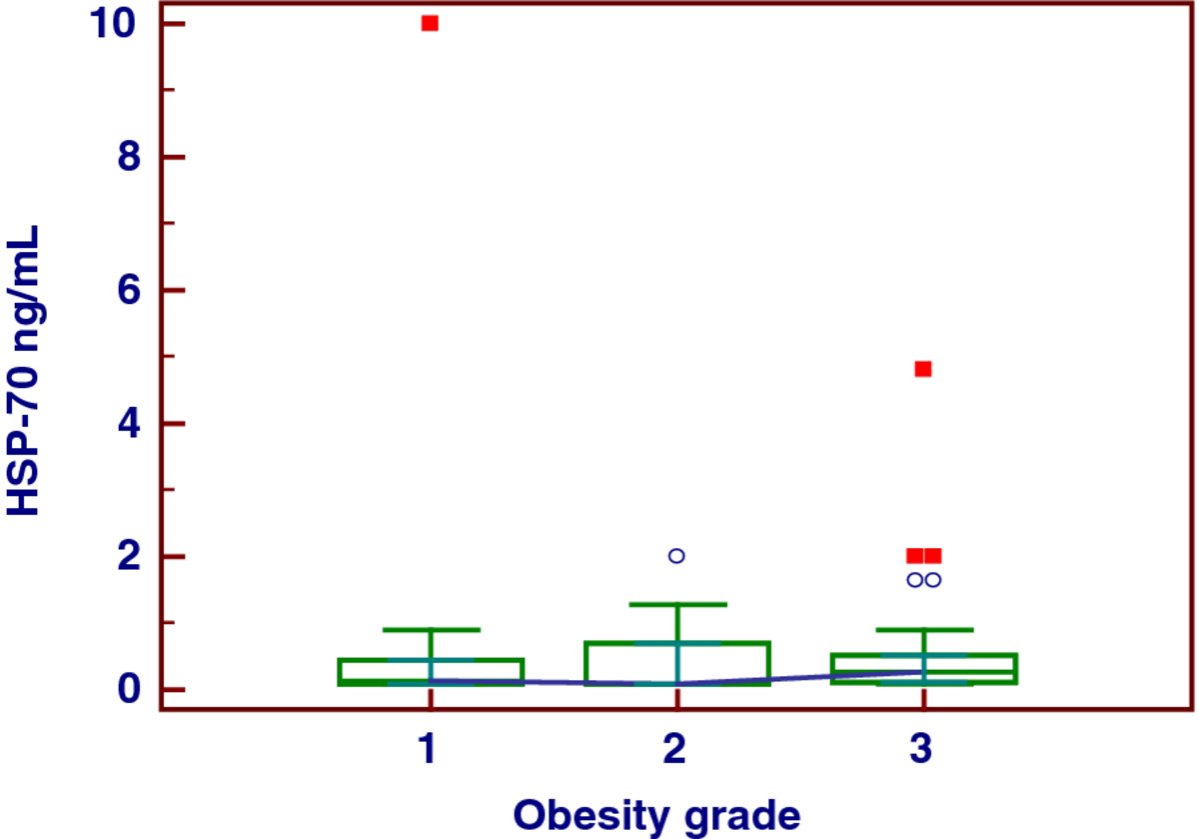
The behavior of heat shock protein-70 in patients with different severity of obesity.

**Figure 4:**
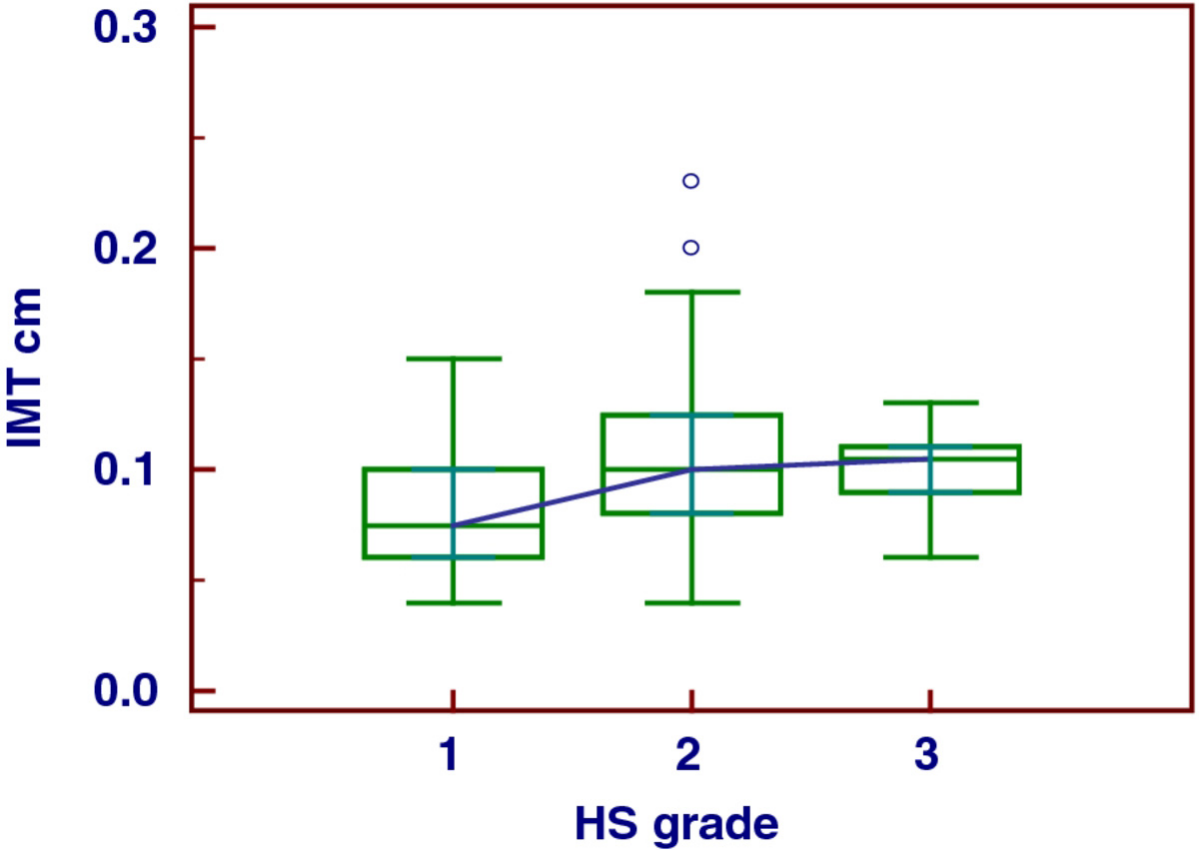
The behavior of carotid intima media thickness in patients with different severity of hepatic steatosis.

**Figure 5:**
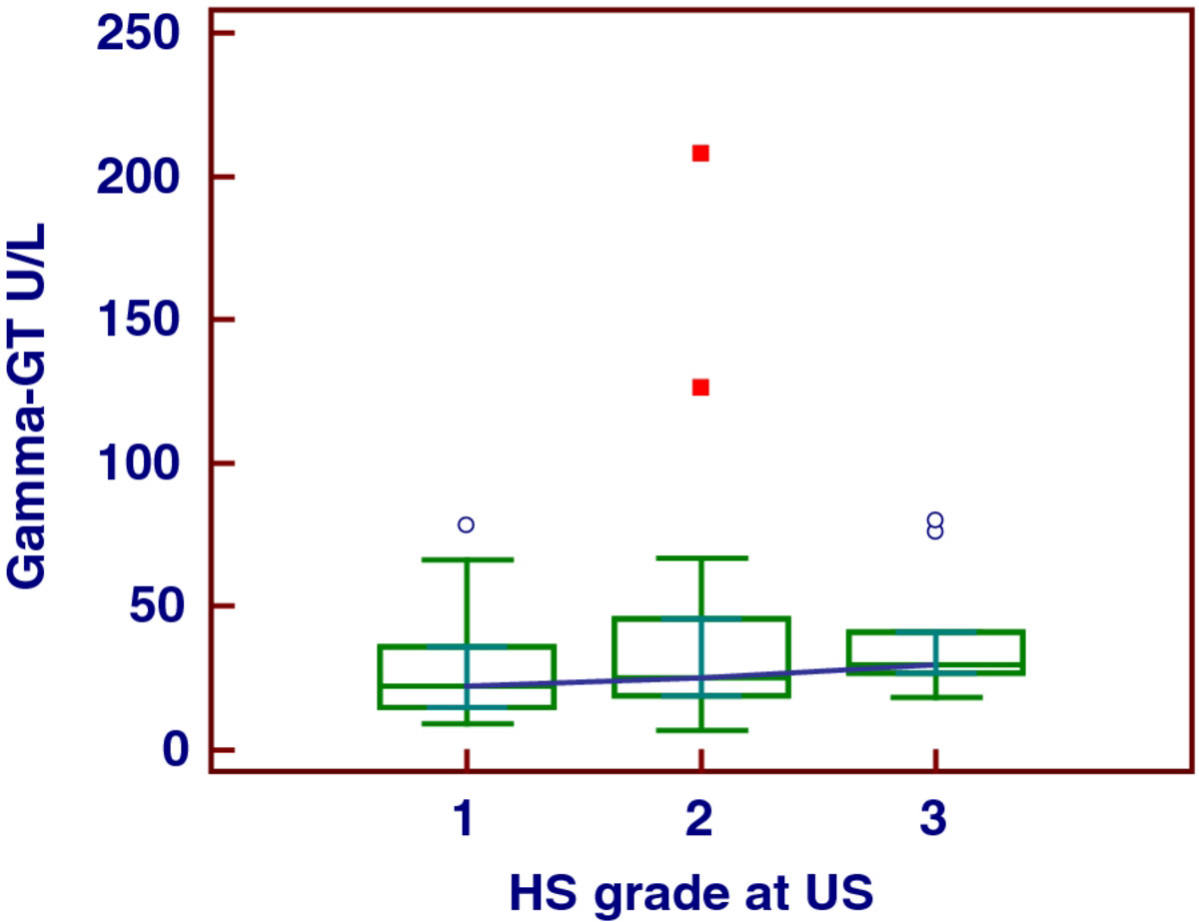
The behavior of gamma-glutamyl transferase in patients with different severity of hepatic steatosis.

The major part of our obese population evidenced HS of moderate grade and was characterized by important visceral adiposity, scarce hepatic cytolysis, increased IMT and progressively increasing frequency of MS throughout the severity of HS, Tables 1, 2. The median serum concentration of HSP-70 in our series of obese patients with NAFLD was clearly lower than that in controls, i.e., 0.13 (IQR = 0.1-0.54) ng/mL versus 1.09 (IQR = 0.81-2.33) ng/mL, *p *< 0.001, Figure 1. There was no significant gender difference when evaluating the serum concentrations of this chaperone, M-W *U *test, *p *= 0.52. At the sub-groups analysis there was a significant distinction in HSP-70 levels between the patients with the HS mildest grade and the more severe one, i.e., grade 1 versus grade 3, ANOVA K-W, post-hoc analysis Conover-Inman test, *p *= 0.03. Indeed, this difference was partially dimmed by some degree of overlap, Figure 2, and was lost when HSP-70 values were adjusted for age, BMI and WC (*p *= 0.60, 0.15, and 0.43, respectively, ANCOVA). The median concentration of HSP-70 in the three groups of obesity was equivalent, ANOVA K-W *p *= 0.23, Figure 3, also when it was adjusted for the grade of HS (ANCOVA, *p *= 0.41). At the sub-groups analysis there was a significant difference in IMT only between the patients with the HS mildest grade and the moderate one, i.e., grade 1 versus grade 2, but burdened by an evident overlap, ANOVA K-W, post-hoc analysis Conover-Inman test, *p *= 0.02, Figure 4. The γ-GT activity, characterized by the major part of values falling into the upper quartile of normal range and a higher median in males than in females (M-W *U *test, *p *= 0.049), was comparable in patients with a high grade and in those with a low grade of HS, ANOVA K-W, *p *= 0.1, Figure 5. The concentrations of HDL and ferritin were similar throughout the three subgroups characterized by increasing HS severity (*p *= 0.16 and 0.35). As expected, a trend of significance of the levels of triglycerides (*p *= 0.07) was clear when passing from the group with the least severe form of HS to the most severe one. SLD and much better CRP showed a progressive increase throughout the severity of HS, ANOVA K-W, *p *= 0.06 and 0.002, respectively. Patients with a hepatic fat deposition of severe/moderate grade had more features of MS respect to those with a light grade (Pearson's chi square, *p *= 0.002).

**Table 1:** Anthropometric, clinical and laboratory data of the whole population

Patients	HS at US	HS at US	HS at US	p
Group	score 1	score 2	score 3	
n	52	128	20	
Age Median years	45	48	49.5	0.27
(IQR)	(25-51)	(37.5-53.5)	(35-55)	
BMI Median	37.7	43	49.2	0.005□
(IQR)	(33.6-40)	(38-46.8)	(44-55)	
Groups' difference	1 from 2	1 from 3	2 from 3	
WC (f) Median cm	110	124	130	0.003□
(IQR)	(104-118)	(118-131)	(124-138)	
Groups' difference	1 from 2	1 from 3	2 from 3	
WC (m) Median cm	116	127	138	0.01□
(IQR)	(110-125)	(123-136)	(134-148)	
Groups' difference	1 from 2	1 from 3	2 from 3	
HOMA Median	2.3	3.1	4.08	0.0037□
(IQR)	(1.18-3.05)	(1.97-4.58)	(3.37-5.89)	
Groups' difference	1 from 2	2 from 3	3 from 1	
Total Cholesterol	201	180	190	0.2
Median mg/dL	(49-222)	(156-210)	(165-205)	
(IQR)				
HDL Cholesterol (f)	54.1 ± 11.5	46.6.1 ± 20.2	38.75 ± 16.6	0.047•
m±SD mg/dL	1 from 2	2 from 1		
Groups' difference				
HDL Cholesterol (m)	39.37 ± 16.5	41.9 ± 13.2	45.6 ± 16.6	0.56
m±SD mg/dL				
Ferritin (f)	46.2 ± 22.4	61.7 ± 41.1	74.5 ± 22.4	0.43
m±SD ng/mL				
Ferritin (m)	197.9 ± 49.6	221 ± 121.7	164.8 ± 23.2	0.72
m±SD ng/mL				
Triglycerides	129 ± 89	151 ± 79	204 ± 121	0.07
m±SD mg/dL				
SAT cm (f)	2.6 (1.4-2.8)	2.9 (2.6-3.6)	3.5 (3.2-4.3)	0.015□
Groups' difference	1 from 2	2 from 1	3 from 1	
SAT cm (m)	1.9 (1.6-2.4)	2.3 (1.9-2.7)	3.5 (2.2-3.7)	0.035*
Groups' difference	1 from 2 and 3	2 from 1 and 3	3 from 1 and 2	
VAT cm (f)	4.5 (4-6.1)	7.1 (6.1-8)	8.2 (8-8.7)	0.001□
Groups' difference	1 from 2	2 from 1	3 from 1	
VAT cm (m)	6.3 (5.6-6.7)	9.2 (7.8-11)	11.5 (9.2-15)	0.0001*
Groups' difference	1 from 2 and 3	2 from 1 and 3	3 from 1 and 2	
SBP mm Hg	125 (120-140)	130 (120-140)	140 (130-160)	0.0002*
Groups' difference	1 from 2 and 3	2 from 1 and 3	3 from 1 and 2	
DBP mm Hg	85 (80-90)	80 (80-90)	80 (80-95)	0.2
SLD mm	108 (95-118)	111 (100-121)	122 (110-140)	0.06
CRP mg/L	0.3 (0.2-07)	0.8 (0.6-1.5)	1.6 (0.7-2.5)	0.002*
Groups' difference	1 from 2 and 3	2 from 1 and 3	3 from 1 and 2	
Fibrinogen mg/dL	290 (240-320)	300 (260-350)	290 (250-400)	0.56
γ-GT U/L	22 (15-36)	25 (19-45.5)	29.5 (27-41)	0.1
ALT U/L	26 (19-40)	30 (27-39)	34 (30-46)	0.17
T2D yes/not	12/40	34/94	7/13	0.33
IFG yes/not	15/37	37/91	9/11	0.42
Hypertension	16/36	47/81	12/8	0.004
yes/not				
MS yes/not	20/32	70/58	13/7	0.002
IMT Median cm	0.08 (0.06-0.11)	0.11(0.09-0.13)	0.12 (0.10-0.13)	0.02#
Groups' difference	1 from 2	2 from 1		

HS, hepatic steatosis; *BMI *body mass index; *WC *waist circumference; *HOMA *homeostasis model assessment of insulin resistance; *SAT *subcutaneous adipose tissue at ultranonography (US), *VAT *visceral adipose tissue at US; *BSP *blood systolic pressure; *DBP *diastolic blood pressure; *SLD *spleen longitudinal diameter at US; *CRP *c reactive protein; *γ-GT *gamma glutamyl transferase; *ALT *alanine aminotransferase; *T2D *type 2 diabetes mellitus; *IFG *impaired fasting glucose; *MS *metabolic syndrome; *IMT *intima media thickness

**Table 2:** Relationship between obesity and hepatic steatosis

Hepatic Steatosis Grade	HS 1	HS 2	HS 3	Total
Obesity Grade 1	20	10	0	30
Obesity Grade 2	20	38	0	58
Obesity Grade 3	12	80	20	112
Total	52	128	20	200

Frequencies of obese patients throughout the grades [1-3] of HS, hepatic steatosis

### Associations & prediction

At univariate analysis the severity of HS did not predict HSP-70 levels (β = -0.03, *p *= 0.7, linear regression).

At multivariate analysis, among the analyzed variables, HSP-70 was predicted only by the serum HDL levels in our obese population, i.e., (β = 0.31, *p *= 0.003, multiple regression, backward stepwise selection).

At univariate analysis, IMT was modestly predicted by the severity of HS (β = 0.22, *p *= 0.03, linear regression), but not by HSP-70 levels (β = 0.16, *p *= 0.24, linear regression). At multivariate analysis IMT was strongly predicted by age, sufficiently well by VAT and discreetly by HOMA (β = 0.50, *p *< 0.0001, β = 0.30, *p *= 0.01 and β = 0.18, *p *= 0.048 respectively, multiple regression backward stepwise selection), but not by IMT. The severity of HS was predicted by VAT, SAT and moderately by HOMA (β = 0.50, *p *< 0.0001 and β = 0.27, *p *= 0.001 and β = 0.18, *p *= 0.024, respectively), multiple regression, backward stepwise selection. The γ-GT activity was well predicted by ALT activity and serum triglycerides concentration and moderately age (β = 0.31, *p *= 0.001, β = 0.27, *p *= 0.002 and β = 0.225, *p *= 0.017, respectively, multiple regression, backward stepwise selection). As expected, γ-GT activity mildly correlated to HOMA (Spearman's rho = 0.28, *p *= 0.043). Mostly interesting, γ-GT activity well predicted SBP (β = 0.30, *p *= 0.02, linear regression). SLD was well predicted by HOMA and SBP (β = 0.20, *p *= 0.04 and β = 0.23, *p *= 0.035, respectively), multiple regression backward stepwise selection. The severity of HS was not predicted by ALT and γ-GT activity (β = 0.14, *p *= 0.12 and β = 0.12 *p *= 0.18, respectively, linear regression). CRP levels were forecasted by BMI (β = 0.23, *p *= 0.02, linear regression). When analyzing the type of adiposity, surprisingly, CRP was predicted by SAT but not VAT (β = 0.36, *p *= 0.0001 and β = 0.135, *p *= 0.18, respectively, linear regression). Accordingly, the factor analysis revealed as hidden relationships that age was strongly related to IMT but not to HS severity, that HS correlated to adipose tissue as well as inflammation (CPR) and finally that the enzymatic activity of ALT and γ-GT, triglycerides and ferritin levels shared same behavior, Table 3. The intra/inter-observational variability of the US estimations was not significant, mean difference = 1.9, 2.1, 2.5, 2.3 and 1.7%, and 2.7, 4.8, 4.6, 4.4 and 3.2% for the bright liver, VAT, SAT, SLD and IMT, respectively, with a ρ_c _of 0.91.

**Table 3:** Factor analysis

	1	2	3
HSP-70	0.068	-0.056	-0.35
AGE	*0.817*	-0.005	-0.056
BMI	0.005	*0.83*	0.04
Waist Circumference	0.15	*0.78*	0.21
Total Cholesterol	0.19	-0.16	-0.25
HDL-Cholesterol	-0.04	-0.02	-0.72
Triglycerides	0.11	0.10	*0.559*
ALT	-0.12	-0.050	*0.56*
GGT	0.27	-0.03	*0.52*
CRP	-0.004	*0.480*	-0.078
Fibrinogen	-0.22	0.24	0.08
Ferritin	0.276	-0.18	*0.51*
Sistolic Blood Pressure	0.38	0.22	0.17
Dastolic Blood Pressure	0.25	0.07	0.285
Hepatic Steatosis	0.25	*0.625*	0.28
HOMA	0.006	0.325	0.49
SAT	-0.275	*0.69*	-0.2
VAT	0.41	*0.575*	0.43
SLD	-0.27	0.08	0.29
IMT-R	*0.80*	0.04	0.095
IMT-L	*0.77*	0.001	0.01
**Percentage of variance explained**			
	13.2	14.5	12.8

Factor analysis (varimax rotation) showing hidden associations, the values of which are evidenced in italics. The critical value was calculated by doubling Pearson's correlation coefficient for 1% level of significance (5.152)/square root of patients (200) minus 2, i.e., 0.366. HSP-70, heat shock protein-70; BMI, body mass index; HOMA, homeostasis model assessment of insulin resistance; SAT, subcutaneous adipose tissue at ultranonography (US), *VAT *visceral adipose tissue at US; *SLD *spleen longitudinal diameter at US; *CRP *c reactive protein; *GGT *gamma glutamyl transferase; *ALT *alanine aminotransferase; *IMTL *intima media thickness of left carotid; *IMTR *intima media thickness of right carotid

## Discussion

Researchers have been recently engaged in a seemingly endless debate about the independent role of NAFLD in moderately increasing the risk of early atherosclerosis as assessed by some Authors [25,26]. One hypothesis could be a direct link between NAFLD and dyslipidemia, endothelial dysfunction, or oxidative stress [27]. In contrast, other researchers demonstrated that NAFLD was not associated with IMT in people with a high prevalence of type 2 diabetes mellitus [28,29]. Among metabolically healthy people, the NAFLD-IMT association was not significant after adjusting for cardiovascular risk factors [30]. HS cannot predict cardiovascular morbidity and mortality in patients with established CAD [31]. Because the severity of HS at US does not predict increased IMT when other risk factors more prominent are included in the multivariate analysis (age, visceral adiposity and IR), we can infer that HS, prevalently of moderate grade as evident in our series, plays a scarce role in determining atherosclerosis. This aspect is reinforced by the lack of association between spleen volume, strictly linked to NAFLD severity [14], and IMT and particularly by the lack of IMT increase throughout the severity of HS, meanwhile the MS stigmata showed a completely different pattern. Another observation in favor of the scarce role played by HS in the atherosclerosis process of our patients is that the entity of visceral adiposity predicted increased IMT but not the HS grade. Mostly interesting, γ-GT activity was intensively related to ALT activity, highlighting its hepatic origin or whatever its strict link. Finally, SLD showed a good relationship to SBP (increased cell adhesion molecules such as E-selectin, vascular cell adhesion molecule-1, and intracellular adhesion molecule-1 expression in splenocytes?). Similarly, researchers are struggling whether NAFLD, determining local IR, could affect systemic IR. Recent results reveal an unexpected role for hepatic IL-6 signaling in insulin action and resistance to limit hepatic inflammation and to protect from local and systemic IR [32]. In contrast, the disruption of insulin signaling in the liver is more relevant to whole body glucose homeostasis than its disruption in adipose tissue and muscle [33]. In addition, hepatic insulin signaling regulates the secretion of very low density lipoprotein and thus lipotoxicity and atherogenesis [34]. Taking into account that IR was particularly present in our series but HS severity modesty predicted HOMA values, hepatic IR does not seem to contribute to peripheral IR. An early sign of atherosclerosis is hypertrophy of the arterial wall [35], even though circulating HSP-70 has atheroprotective effects [36]. Accordingly, a strict association between HSP-70 and HDL levels [37] was found, even though an inverse correlation between HSP-70 and IMT was lacking. Data from our series showed that γ-GT activity, although moderately correlated to IR, was not different on the basis of the HS severity. Interestingly, high γ-GT levels were clearly associated with hypertension, specifically with systolic values (SBP), but not with increased IMT. The association between CRP and early atherosclerosis is documented by numerous works and CRP is recently proposed as cause of the latter [38]. Our data point out on the strong correlation between subcutaneous but not visceral adiposity and CRP levels according to previous findings [39].

As limitations, we stress the followings. It has previously been shown that in dyslipidaemic and obese individuals there are elevated titres of HSPs antibodies [40], perhaps affecting the serum levels HSPs. In this case, ageing should cause an increase of the circulating levels of HSP-70 and not a decrease of the same ones [8]. The assessment of HS severity could have been more precise with liver biopsy even though US, as imaging tool, is reliable for large scale studies [41].

## Competing interests

The Authors declare that they have no competing interests

## Authors' contribution

GT conceived the study, conducted the statistical analysis and drafted the manuscript. CF and SS carried out the clinical investigation. GT, SG and MT performed sonographic procedures. EG, DC and FS were responsible for laboratory data. AC, DC, SS, FP and FC critically revised the manuscript. All authors read and approved the final manuscript.

## Financial support

This research was supported by departmental funds of Federico II University Medical School
